# Metalation: nature’s challenge in bioinorganic chemistry

**DOI:** 10.1007/s00775-020-01790-3

**Published:** 2020-04-24

**Authors:** Nigel J. Robinson, Arthur Glasfeld

**Affiliations:** 1 Department of Bioscience and Department of Chemistry, University of Durham, Durham, UK; 2 Chemistry Department, Division of Mathematical and Natural Sciences, Reed College, Oregon, USA

## Abstract

The association of proteins with metals, metalation, is challenging because the tightest binding metals are rarely the correct ones. Inside cells, correct metalation is enabled by controlled bioavailability plus extra mechanisms for tricky combinations such as iron and manganese.

In this issue [1], Grāve, Högbom and colleagues address a tremendously important challenge: How do proteins acquire the correct metals? This is important because almost a half of enzymes are estimated to require metals [2, 3]. This is a challenge because nascent proteins offer negligible steric selection. Under these circumstances, the binding of divalent metals ions will follow the Irving–Williams series [2, 4, 5]. Where there is a metal delivery protein the challenge is (to some degree) amplified rather than solved, that is, the correct metal must also now be acquired and supplied by the delivery protein, somehow.

Mg^II^ < Mn^II^ < Fe^II^ < Co^II^ < Ni^II^ < Cu^II^ (Cu^I^) > Zn^II^.

The Irving–Williams series of divalent metal-binding preferences [4]. Cuprous ions also bind tightly and note reversal of the less-than sign after copper.

Divalent ions of manganese and of iron are towards the weaker binding end of the Irving–Williams series and so there is a risk that their binding sites could become mis-metalated with tighter binding divalent cobalt, nickel, zinc or copper ions (or monovalent copper which is thought to predominate in the cytosol and bind tightly, especially to sites containing thiols). Mis-metalation need not be structurally conservative, because an incorrect metal may use only a subset of ligands, or recruit additional ligands, or distort the geometry within a flexible nascent protein. Therefore, it could reasonably be argued that mis-metalation often involves different but competing metal sites. However, the biological challenge is to avoid mis-metalation at some locus within a nascent protein, regardless of the conformation of the aberrant site. Crucially, cells maintain the availabilities of these different elements to the inverse of the Irving–Williams series with Mn(II) and Fe(II) at greater availabilities than all of the above listed tighter binding metals (Fig. 1) [6]. Thus, under the conditions of metal availability that occur inside cells correct metalation becomes a much more modest challenge.

**Fig. 1:**
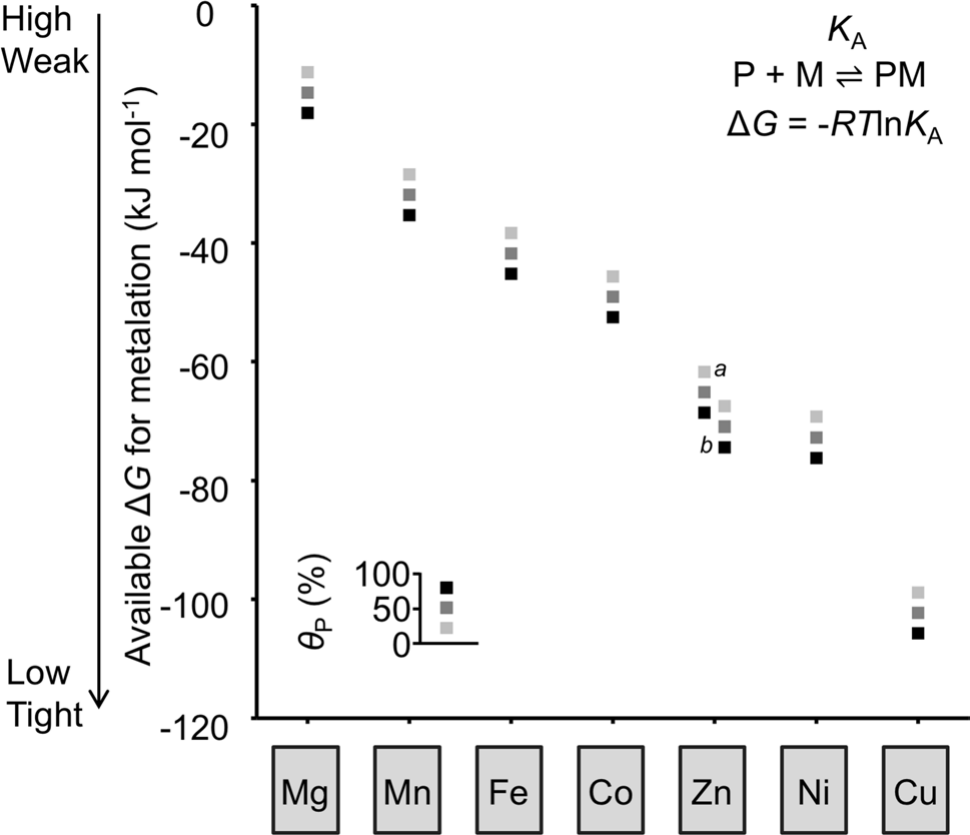
The availabilities of metals inside a bacterial cell are set to the inverse of the Irving–Williams series. Metal availabilities have been determined from the sensitivities of DNA-binding, metal-sensing, transcriptional regulators (adapted from reference [6]). The boxes represent the free energies for metal complex formation with proteins that are 80%, 50% or 20% (black to light grey, respectively) metalated in an ideal cell where the metal-sensors are at the mid-points of their dynamic ranges, using the standard equation as shown. Proteins can acquire metal via rapid ligand-exchange reactions with molecules that buffer availabilities to the determined values, only when the free energy gradient is favourable

Iron and manganese are used by about 8% and 6% of all enzymes, respectively, representing about 18% of the metalloenzymes without dedicated delivery systems: albeit other sets of iron enzymes bind pre-assembled cofactors and do have delivery systems [7]. Fe(II) and Mn(II) are proximal in the Irving–Williams series and the dynamic ranges for the availabilities of Mn(II) and Fe(II) inside some cells partly overlap (Fig. 1). There is evidence that mis-metalation of manganese sites with iron (and the reverse) can occur within cells [8–10]. Manganese superoxide dismutase (SOD) in *E. coli* is commonly mis-metalated with iron and inactive. In response to oxidative stress, a sensor triggers expression of manganese import, manganese availability rises and nascent SOD becomes correctly populated with manganese [8–10]. The question addressed by Högbom and colleagues [1], concerning discernment between manganese and iron, is especially pertinent.

An inherent specificity of a class Ib ribonucleotide reductase (RNR) for manganese over iron is intriguing, because, in the first instance, this seemingly defies the Irving–Williams series [1]. In bacteria, two classes of RNR show divergent functional requirements for metal ions: class Ia enzymes are functionally dependent on iron while class Ib enzymes are manganese-dependent [11]. Past investigations suggest that the oxidation of Fe(II) or Mn(II) in the metal-binding sites plays a dominant role in metal ion selectivity. In vitro, Fe(II) can be autoxidized without catalysts to Fe(III) once it binds to the class Ia RNR site [12], though inside the cell Fe(II) oxidation is assisted by an accessory ferredoxin [13]. Mn(II) does not autoxidize in class Ib RNR in vitro, and in vivo class Ib RNR proteins are co-expressed with a flavodoxin-like protein (NrdI) that functions as a catalyst for the oxidation of Mn(II) to Mn(III) in the RNR metal-binding site [14]. Note also that class Ib RNRs typically function in cellular environments with high manganese concentrations. For both classes, following oxidation, the dissociation rate of iron or manganese is presumably much reduced.

Given the above understanding of how specificity arises in class Ia and Ib RNRs through oxidation and the involvement of accessory factors in that process [13, 14], the finding that class Ib RNRs can exhibit inherent selectivity for Mn(II) without any accessory factors is intriguing, although oxidation to Mn(III) likely still plays a part [1]. The inherent flexibility of amino acid side chains is a hurdle to generating binding sites that show steric preference for a single metal ion. Indeed, the metal-binding site(s) of the class Ib RNR from *E. coli* show different geometries when occupied by Mn^2+^ or Fe^2+^ (Fig. 2) [15]. However, in such di-metal sites as described for both class Ia and Ib ribonucleotide reductases, there is scope for steric selection such that the order of binding preferences can vary from the Irving–Williams series. The first metal to bind can pre-organise the second site to a preferred geometry [16]. Positive and negative cooperativity between the two sites in a binuclear complex could be a powerful mechanism for generating selectivity counter to the trend of the Irving–Williams series. For instance, if Fe(II) binds to either site with geometry that lowers affinity at the second site, then the binuclear complex is unlikely to form. However, if the first Mn(II) binds in such a way as to enhance Mn(II) binding to the second site, the binuclear Mn(II) form of the enzyme could dominate, even if Fe(II) is capable of competing with Mn(II) for either individual site.

**Fig. 2:**
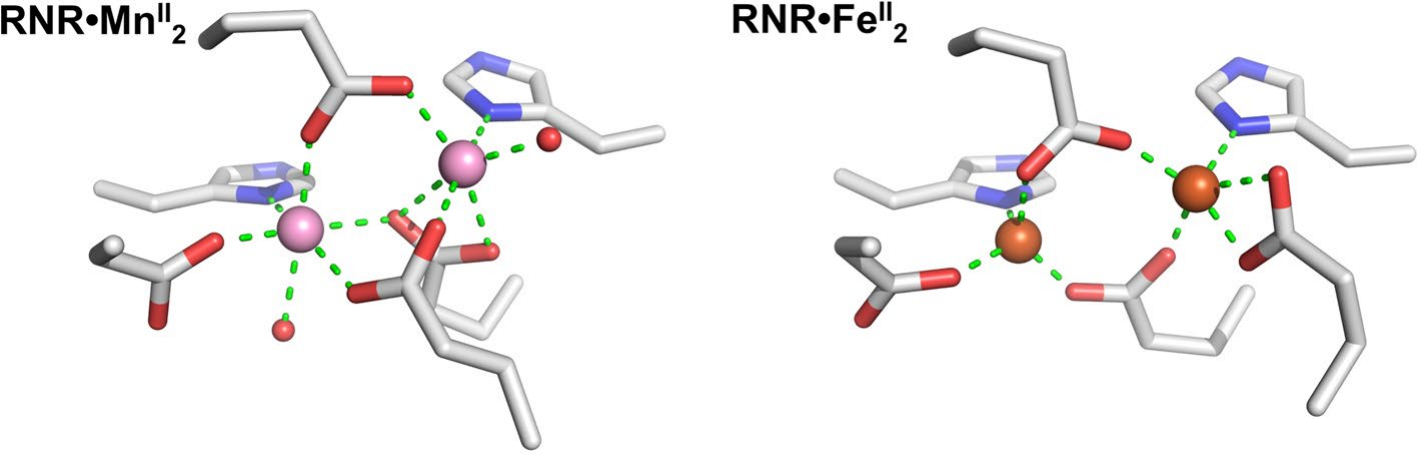
The metal-binding site of the class Ib RNR of *E. coli* occupied with Mn^2+^ (left) and Fe^2+^ (right). Ligating side chains and water molecules are shown for bound Mn^2+^ (pink spheres) and Fe^2+^ (orange spheres) along with green dashes to indicate bonding interactions (< 2.3 Å). Note that the coordination geometry for Mn^2+^ is hexacoordinate in each site, while for iron the same set of residues contribute to tetra- and pentacoordinate geometries (PDB 3N37 and 3N38 using PyMOL, Schrödinger, LLC)

There will only have been evolutionary selection pressure for proteins to show sufficient discrimination to obtain the correct metal under the conditions of metal availability that prevail in nature. Thus, intracellular metalation substantively bypasses the challenge presented by the Irving–Williams series, because the less competitive metals are at higher availabilities than the more competitive ones. But herein, we see how this process is augmented by mechanisms that, for example, introduce steric selection and changes in oxidation state for residually challenging combinations of metals such as iron and manganese [1]. The thermodynamic framework in Fig. 1 presents the alluring prospect of being able to evaluate the relative contributions of such mechanisms to the correct metalation of half of the reactions of life.
